# Saffron as an antidote or a protective agent against natural or chemical toxicities

**DOI:** 10.1186/s40199-015-0112-y

**Published:** 2015-05-01

**Authors:** Bibi Marjan Razavi, Hossein Hosseinzadeh

**Affiliations:** Targeted Drug Delivery Research Center, Department of Pharmacodynamy and Toxicology, School of Pharmacy, Mashhad University of Medical Sciences, Mashhad, Iran; Pharmaceutical Research Center, Department of Pharmacodynamy and Toxicology, School of Pharmacy, Mashhad University of Medical Sciences, Mashhad, Iran

**Keywords:** Saffron, *Crocus sativus* L, Crocin, Crocetin, Safranal, Antidote, Protective, Natural toxin, Chemical toxin

## Abstract

Saffron (*Crocus sativus*) is an extensively used food additive for its color and taste. Since ancient times this plant has been introduced as a marvelous medicine throughout the world. The wide spectrum of saffron pharmacological activities is related to its major constituents including crocin, crocetin and safranal. Based on several studies, saffron and its active ingredients have been used as an antioxidant, antiinflammatory and antinociceptive, antidepressant, antitussive, anticonvulsant, memory enhancer, hypotensive and anticancer. According to the literatures, saffron has remarkable therapeutic effects. The protective effects of saffron and its main constituents in different tissues including brain, heart, liver, kidney and lung have been reported against some toxic materials either natural or chemical toxins in animal studies.

In this review article, we have summarized different in vitro and animal studies in scientific databases which investigate the antidotal and protective effects of saffron and its major components against natural toxins and chemical-induced toxicities. Due to the lake of human studies, further investigations are required to ascertain the efficacy of saffron as an antidote or a protective agent in human intoxication.

## Background

*Crocus sativus* L., is a perennial herb which belongs to the Iridaceae family and is cultivated in Azerbaijan, France, Greece, India, Iran, Italy, Spain, China, Morocco, Turkey, Egypt, and Mexico [1]. Saffron, the dried stigma of the *C. sativus*, has been extensively used as a spice and food colorant because of its color and taste [2].

Saffron contains more than 150 chemicals agents [3]. Among which three main components of saffron are responsible for its pharmacological effects including: crocins, the principle coloring agent (mono and diglycosyl esters of a polyene dicarboxylic acid, named crocetin) [4], the glycoside picrocrocin which is a precursor of safranal and responsible for its bitter taste and safranal, a monoterpen aldehyde which is the deglycosylated form of picrocrocin and is responsible for the characteristic aroma of saffron [5,6].

In folk medicine, saffron has been believed to have several properties such as antispasmodic, eupeptic, anticatarrhal, nerve sedative, carminative, diaphoretic, expectorant, stimulant, stomachic and aphrodisiac [7,8]. Moreover, modern pharmacological studies have demonstrated that saffron and its constituents have a wide spectrum of activities including antioxidant [9], anticonvulsant [10-12], antidepressants and anxiolytics [13-16], antinociceptive and anti-inflammatory [17,18], memory enhancers [19-21], antitussive [22], reducing withdrawal syndrome [23], improving male erectile dysfunction [24], hypotensive [25-27], anticancer [28,29] and antisolar [30,31].

Furthermore according to the literature, the protective effects of saffron as well as its active components in different tissues including brain [32], heart [33], liver [34], kidney [35], lung [36] and etc have been reported against some toxic materials.

## Methods

In this review article, we have discussed different studies in scientific databases including Scopus, MEDLINE and Web of Science databases and local references, which investigate the antidotal and protective effects of saffron and its major components against natural toxins and chemical-induced toxicity. Studies were identified through electronic databases from their inception up to October 2014. The keywords for the search were: *Crocus sativus*, saffron, crocin, safranal, crocetin, antidote, natural toxin, chemical toxin and protective effects.

### Natural toxins

Documents have been shown saffron and its main components exhibit antidotal effects against some natural toxins including snake venoms [37], aflatoxins [38], lipopolysaccharides (LPS) [39] and 3-nitropropionic acid (3- NP) [40]. These effects might be due to their antioxidant [41], anti-inflammatory [37] and antiapoptotic [37] effects (Table 1).

**Table 1:** **Antidotal effects of saffron and its main constituents against natural toxins**

**Toxin**	**In vitro/In vivo**	**Constituents**	**Results**	**Ref. NO**
Snake venom	Swiss albino male mice	Crocin	Suppression of oxidative stress, hematological alteration and pro inflammatory cytokine levels	[37]
Snake venom	Isolated platelet	Crocin	Inhibition of oxidative stress and platelet apoptosis	[41]
Snake venom	Isolated neutrophils	Crocin	Inhibition of oxidative stress and neutrophil apoptosis	[42]
AflatoxinB1	Male Wistar rats	Crocetin (0.1 mg/day/rat)	Reduction of AST, ALT, AlP, and γ-GGT, Elevation of GSH, Reduction of the formation of hepatic AFB_1_-DNA adducts	[38]
AflatoxinB1	Female Sprague-Dawley rats	Crocin dyes (50 mg/kg/day, 3 days)	Reduction of AST, ALT, AlP, γ-GGT and LDH	[47]
Lipopolysaccharide	mice	Crocetin (50 and 100 mg/kg, gavage) for 24 hr	Reduction of lung edema, Increase in SOD, Decrease in MPO, Attenuation of mRNA and protein expressions of IL-6, MCP-1, TNF-α, P-IκB and NF-κB	[39]
Lipopolysaccharide	RAW 264.7 macrophages	Crocin	Suppression of iNOS induction of HO-1 expression via Ca2+/calmodulin-CAMK4-PI3K/Akt-Nrf2	[45]
Lipopolysaccharide	Rabbit	Crocetin	Improve of DIC-related haemostatic indices such as platelet blood counts, blood plasma fibrinogen and protein C concentration, Amelioration of DIC-associated disease and fibrin deposition in the glomeruli	[43]
Lipopolysaccharide	RAW 264.7 macrophages	Crocin	Inhibition of the PGE(2) products, Prevention of NF-kappaB p50 and p65 subunits	[44]
3-nitropropionic acid	Isolated striatal synaptosomes	Saffron extract (1 mg/kg/day, for 5 days, IP.)	Decrease of lipid peroxidation, Improve of mitochondrial function	[40 ]

### Snake venoms

It is established that crocin could neutralize oxidative stress and hematological complications induced by viper venoms. The pre-incubation of crocin with venom (1:10; venom: crocin, w/w) at 37°C for 10 min), suppressed the venom-induced oxidative stress, hematological alteration and pro inflammatory cytokine levels in Swiss albino male mice [41]. Furthermore, the inhibitory effect of crocin on viper venom-induced platelet and neutrophil apoptosis has been shown in other studies [37,42]. Crocin ameliorated the *Vipera russelli* venom-induced apoptotic events such as the generation of endogenous ROS, mobilization of intracellular calcium, depolarization of mitochondrial membrane, cytochrome c release, caspase activation, phosphatidylserine externalization and DNA damage [37,42].

### Lipopolysaccharide (LPS)

Based on the documents, saffron active constituents could suppress LPS-(endotoxin derived from gram-negative bacteria) induced mice lung injury [39], distributed intravascular coagulation in rabbits (DIC) [43] and LPS-stimulated RAW 264.7 macrophages [44].

Briefly, crocetin (50 and 100 mg/kg, gavage) could reduce the LPS-induced lung edema and histological changes, increased LPS impaired SOD activity, and decreased lung MPO activity. Moreover, crocetin significantly attenuated LPS-induced mRNA and the protein expressions of IL-6, MCP-1, TNF-α, phospho-IκB expression and NF-κB activity [39].

Another study revealed crocetin could improve DIC related haemostatic indices impaired by endotoxin including platelet blood counts, blood plasma fibrinogen and protein C concentration in rabbits [43].

In addition, in vitro studies showed that crocin suppressed the LPS-stimulated expression of iNOS by inducing HO-1 expression via Ca^2+^/calmodulin-dependent protein kinase 4 -PI3K/Akt-Nrf2 signaling cascades [45].

In another study, crocin inhibited the prostaglandin E2 products in LPS-stimulated RAW 264.7. Furthermore, crocin prevented the nuclear translocation of the NF-kappa B p50 and p65 subunits [44].

### Mycotoxins

#### Aflatoxin B1 (AFB1)

AFB1 is an aflatoxin produced by *Aspergillus flavus* and *Aspergillus parasiticus* [46]. Crocetin and crocin were found to possess protective effects against AFTB1 hapatotoxicity and AFTB1 DNA adducts via the reduction of hepatic injury markers (AST, ALT, ALP and γ-GGT) and elevations of hepatic glutathione (GSH) and activities of GST and GSH-Px in animal models [38,47].

#### Nitropropionic acid (3-NPA)

3-NPA is a fungal toxin which is known to affect mitochondria and subsequently leads to ATP depletion and causes neurotoxicity [40]. The protective effect of saffron extract in striatal synaptosomes isolated from the brain of rats exposed to the mitochondrial toxin 3-NPA has been reported. 3-NPA-(20 mg/kg/day, for 3 days, IP.), induced a significant increase in lipid peroxidation and decreased the mitochondrial function in synaptosomal fractions. However, saffron extract (1 mg/kg/day, for 5 days, IP.) decreased lipid peroxidation and improved mitochondrial function through antioxidant property [40].

### Chemical-induced toxicity

#### Protective effects of saffron against chemical-induced hepatotoxicity

Based on the evidences from animal studies, saffron has ability to possess protective effects against hepatotoxicity induced by some materials including beryllium chloride (BeCl2) [48], aluminum chloride (AlCl3) [49], carbon tetrachloride (CCl4) [50], acetaminophen [51], cyclophosphamide [52], diazinon (DZN) [34] and paraquate (PQ) [53] through modulation of antioxidant enzymes [49,52], improvement in structural liver damages [51], reduction in markers of hepatic injury such as AST, ALT, ALP, LDH, GGT, lipid and protein oxidation [48,50-52], alleviation of apoptosis [34], increase in GSH [48] and improvement in lipid dysregulation through ERK1/2 pathway [54].

#### Metals

##### Beryllium (Be)

BeCl2 is a highly toxic material which accumulates in different tissues after absorption. Oral intake through drinking water is a common route of human exposure to Be. Furthermore, workers are exposed to Be containing dusts during the crushing and grinding of ores, and during the processing of Be metal and alloys [55].

It is documented that crocin (200 mg/kg, for 7 consecutive days with BeCl2 or 7 consecutive days before BeCl2, IP.), reduced BeCl2-(86 mg/kg, orally for 5 consecutive days) induced liver toxicity. The increase in MDA and LDH levels, decrease in GSH content and haematological parameters induced by BeCl2 were modulated by crocin [48].

#### Aluminum (Al)

Aluminum (Al) is the third most abundant element in nature [56]. It is a constituent of cooking utensils, medicines, deodorants, and food additives. The sources of Al include corn, yellow cheese, salt, herbs, spices, tea, cosmetics, cookwares, and containers [57].

Shati et al. (2010) revealed saffron and honey minimized the toxic effect of AlCl_3_ in the liver by alleviating its disruptive effect on the biochemical and molecular levels. A significant increase in the cholesterol levels, triglycerides, GGT, ALT, AST, ALP, lipid peroxidation, and glucose were observed in the AlCl_3_ group. However, co treatment of AlCl_3_ with saffron and honey improved the disrupted liver biochemical markers and alleviated the increase of lipid peroxidation [49].

#### Acetaminophen

Acetaminophen (N-acetyl-p-aminophenol) is a widely used drug as an analgesic and antipyretic (Ahmad, 2010). It is a known hepatotoxic in overdose [58]. Omidi et al. (2014) showed that 20 mg/kg of *C. sativus* petals hydroalcoholic extract ameliorated acetaminophen-induced acute liver injury in rats through reducing the levels of AST, ALT and bilirubin, and increased the total protein and albumin. Cell swelling, severe inflammation and necrosis were observed in acetaminophen exposed rats; however in saffron treated rats only mild hepatocyte degeneration was seen [51].

#### Carbon tetrachloride (CCl4)

CCl4 is a solvent which causes liver toxicity by many roots of administration (oral, inhalation, and parenteral exposures) [50]. It has been shown that CCl4-induced fatty degeneration and vacuole formation and increased the levels of ALT and AST in plasma. The aqueous and ethanolic extracts of *C. sativus* stigmas and petals significantly decreased these impairments [50].

#### Cyclophosphamide

Cyclophosphamide is an alkylating agent commonly used as a chemotherapeutic and immunosuppressive drug [59]. A study by Jnaneshwari et al. (2013) exhibited the protective efficacy of crocin against hepatotoxicity induced by cyclophosphamide in Wistar rats. Crocin (10 mg/kg for 6 days, orally) after the administration of a single dose of cyclophosphamide (150 mg/kg, IP.), significantly improved hepatic and antioxidant enzymes, lipid and protein oxidation [52]. Another study showed crocetin significantly elevated GST activity both in the bladder and the liver of mice treated with cyclophosphamide [60].

#### Pesticides

##### Paraquat

It was reported that paraquat (PQ 5 mM), a widely used herbicide, increased leakage of LDH and ALT in rat primary hepatocytes and crocetin (10, 20 μM) significantly suppressed the hepatotoxicity [53].

##### Diazinon (DZN)

DZN is an organophosphate insecticide. In addition to the acetyl cholinesterase inhibition, it can damage tissues via oxidative stress [61]. Lari et al. (2013) indicated that crocin reduced DZN- induced hepatotoxicity through suppression the increase in MDA and attenuation the activation of caspases and reduction the Bax/Bcl-2 ratio [34].

### Protective effects of saffron against chemical-induced cardiovascular toxicity

The protective effects of saffron and its active constituents have been shown against DZN (an organophosphate insecticide) [33], doxorubicin (an antitumor agent) [62] and isoproterenol-(a synthetic non-selective β adrenoceptor agonist) [63] induced cardiac toxicity in previous studies through several mechanisms including modulation of cardiac hemodynamic [63], histopathological and ultrastructural impairments [63], improvement in cardiac markers such as CK-MB [33,64], alleviation of lipid peroxidation [33], suppression of genes involved in cardiac apoptosis and anti-inflammatory effects [33].

#### Diazinon (DZN)

**Figure 1:**
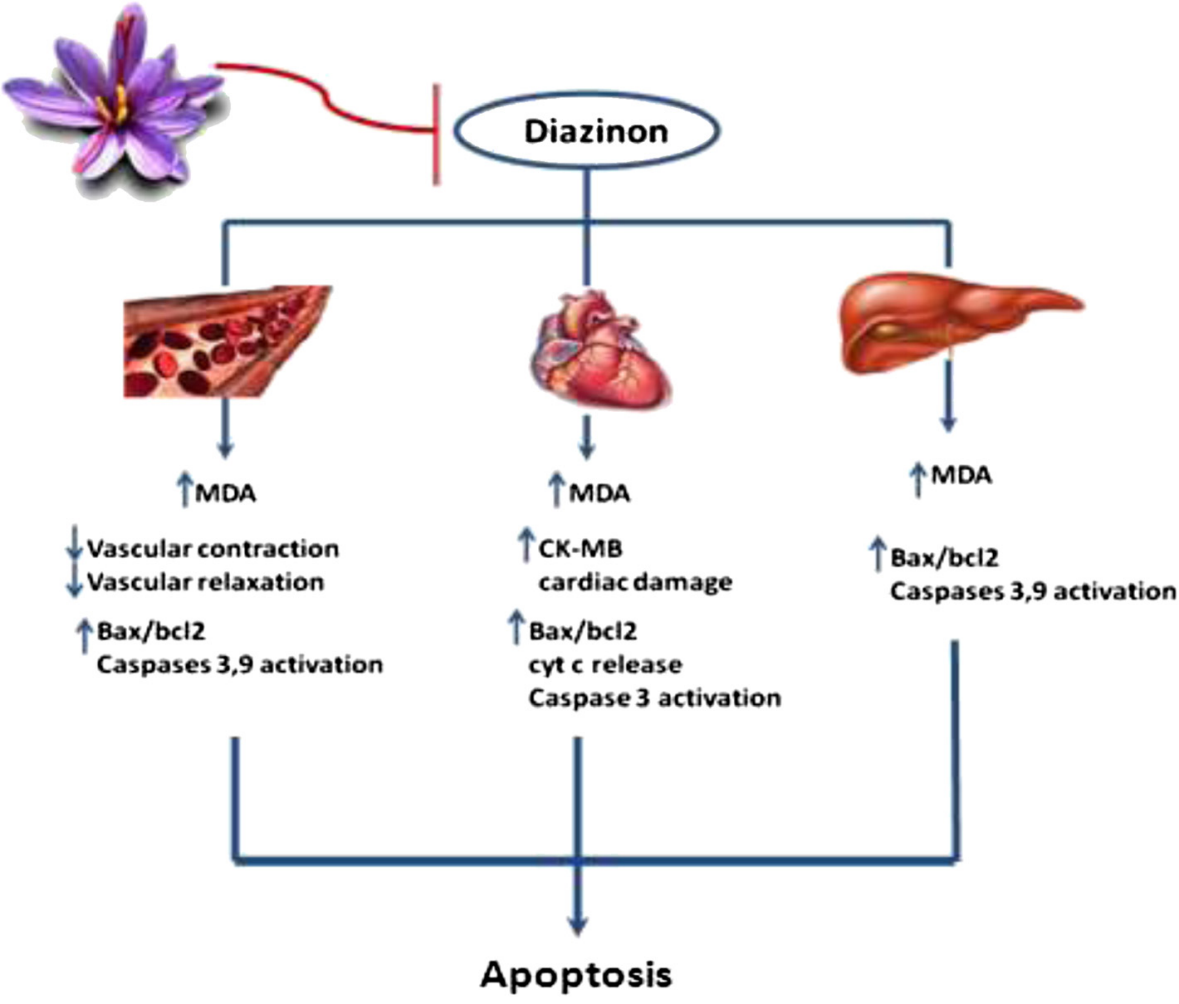
Schematic mechanistic description of saffron against toxicity induced by DZN.

It was reported that DZN-(15 mg/kg, gavage, for 28 days) induced vascular toxicity which may be due to oxidative stress and not to a cholinergic mechanism [65]. Crocin (20 mg/kg, IP., for 28 days) improved toxic effects of DZN via reducing lipid peroxidation and restoring altered contractile and relaxant responses in rat aorta [65]. In addition concurrent administration of crocin and DZN could restore the effects of subchronic DZN administration on systolic blood pressure and heart rate in rats [66]. Besides antioxidant effects, a research has been shown that crocin exhibited protective effects against DZN-induced mitochondrial-mediated apoptosis in heart tissue of rat following subchronic exposure [33] (Figure 1). Furthermore, it was reported the aqueous extract of *C. sativus* (saffron) stigma and its main components, crocin and safranal, prevented DZN-induced enzymes elevation and some specific biomarkers including, CK-MB, TNF-a, 8-iso-prostaglandin F2a and soluble protein-100β in rats [67,68].

#### Isoproterenol

Mehdizadeh et al. (2013) demonstrated saffron or safranal could reduce histopathological damages as well as lipid peroxidation in rat heart tissues and also decreased CK-MB and LDH activities in serum induced by isoproterenol [64]. Similar study showed crocin (20 mg/kg/day) may have cardioprotective effects in isoproterenol-induced cardiac toxicity via modulation hemodynamic, antioxidant, histopathological and ultrastructural impairments [63].

#### Doxorubicin

Doxorubicin is an anthracycline antibiotic which is used as an antitumor agent [69]. It is well known that anthracyclines can induce cardiotoxicity by releasing ROS [62].

To evaluate the effect of saffron against acute myocardium injury by anthracyclines, the model of an isolated rabbit heart was used. ROS was generated by electrolysis of the perfused heart solution and/or generated by perfusion with 30 μM doxorubicin in the presence and absence of 10 μg/ml saffron extracts. ROS generated by two models affects cardiovascular function; it decreased ventricular pressure, heart rate and coronary flow and increased lipid peroxidation while SOD activity decreased. The myocardial architecture was also altered by ROS. Saffron perfused during electrolysis could trap ROS and significantly improved myocardial function [62].

### Protective effects of saffron against chemical-induced genotoxicity

#### Antitumors

It was proved saffron extract could protect against some antitumor agents including cisplatin [70,71], cyclophosphamide [70,71], mitomycin-C [70,71], and methyl methanesulfonate (MMS) [72] in animals via modulation of lipid peroxidation, antioxidants and detoxification systems [70]. Saffron (20, 40 and 80 mg/kg) was orally administered to mice for 5 days prior to these antitumor agents. A significant reduction in lipid peroxidation with an increase in the liver enzymatic (SOD, CAT, GST, GPx) and non-enzymatic antioxidants (GSH) were observed in saffron pretreated animals. Moreover saffron significantly inhibited antitumor drugs-induced cellular DNA damage (strand breaks) as revealed by decreased comet tail length, tail moment and DNA percentage in the tail [70,71].

Hosseinzadeh et al (2007 and 2008) reported the effect of aqueous extract of *C. sativus* stigmas, crocin and safranal on MMS-(120 mg/Kg, IP.) induced DNA damage in different mice organs using the comet assay. The MMS-induced DNA damage (increase in the % tail DNA) (80 mg/Kg) was decreased in some tissues including kidney, lung and spleen in *C. sativus* stigmas aqueous extract, crocin and safranal pretreated mice [72,73].

#### Paraquat (PQ)

Crocetin also decreased genotoxicity induced by PQ. In this study oxyradical generated by PQ caused DNA damage which was evaluated with unscheduled DNA synthesis in rat primary hepatocytes [53].

### Protective effects of saffron against chemical-induced pulmonary toxicity

#### Benzo[a]pyrene

Benzo[a]pyrene is a polycyclic aromatic hydrocarbon isolated from coal tar [36]. It can interact with lipids of membrane and consequently produces free radicals [36]. A study showed that single dose of benzo[a]pyrene (100 mg/kg, IP) could increase the level of ROS and lipid peroxides in lung mitochondria, and reduced the levels of membrane ATPase, GSH and lung mitochondrial enzymes in mice. Furthermore, the level of 8-Hydroxy-2-deoxyguanosine (8-OHdG) in lung DNA of mice was induced by benzo[a]pyrene. Crocetin (20 mg/kg, IP.) protected the structural and functional impairment of lung mitochondria induced by benzo[a]pyrene following 18 weeks treatment (starting from 4th to 22nd week) after the first dose of benzo[a]pyrene [36].

### Protective effects of saffron against chemical-induced neurotoxicity

#### Aluminum

Aluminum is the third most abundant element in nature [36]. It is an accepted neurotoxin implicated in the pathogenesis of neurodegenerative diseases [32]. Linardaki et al. (2013) reported Al intake (50 mg/kg/day in the drinking water for 5 weeks) could cause memory impairment, increase of brain MDA and reduction of GSH content, significant reduction of AChE and BuChE activity, activation of brain MAO isoforms and inhibition of cerebellar MAO-B in mice. Although co-administration with saffron extract (60 mg/kg/day, for the last 6 days, IP.) had no effect on cognitive performance, it reversed significantly the Al-induced changes in MAO activity and the levels of MDA and GSH.

Another study revealed 40 mg/kg/day of A1C1_3_ for 90 days caused a decrease in the AChE activity and enzymatic antioxidant activities in both cerebral hemisphere and cerebellum of rats. Moreover, the expression of A Disintegrin and Metalloprotease, AChE, P53, Bcl-2 and interleukins (IL-4 and IL-12) genes in AlCl_3_ group was changed. Saffron aqueous extract (200 mg/kg/day) attenuated the neurotoxic effects of A1C1_3_ [74].

#### Acrylamide (ACR)

ACR, is an industrial potent neurotoxic agent in human and animals that has been recently found in carbohydrate rich foods cooked at high temperatures [75]. The effect of crocin, on ACR-induced cytotoxicity was evaluated using PC12 cells. The pretreatment of cells with 10-50 μM crocin significantly attenuated ACR cytotoxicity in a dose-dependent manner. Crocin inhibited the down regulation of Bcl-2 and the up regulation of Bax and decreased apoptosis in treated cells. Also, crocin inhibited ROS generation in cells exposed to ACR [76].

### Protective effects of saffron against chemical-induced nephro-or uro-toxicity

#### Antitumors

Cisplatin is an antitumor agent which induces nephrotoxicity via oxidative stress [77]. Naghizadeh et al. (2008) showed blood urea, creatinine, urinary glucose, protein concentrations and oxidative stress markers in crocin treated groups (100, 200 and 400 mg/kg, IP., for 4 consecutive days ) were significantly lower than those of cisplatin (5 mg/kg). Cisplatin caused damage in S3 segment of proximal tubules, whereas no damage was observed in crocin treated rats [77].

Moreover, the administration of cysteine and vitamin E, *C. sativus* and *Nigella sativa* together with cisplatin partly reversed the kidney enzymes impairments induced by cisplatin [78].

Another study revealed crocetin (50 mg/kg) modulated the release of chloroacteldehyde, a urotoxic metabolite of cyclophosphamide in the urine of mice [60].

#### Antibiotics

The nephroprotective activity of saffron against gentamicin and ceftazidime-induced nephrotoxicity has been shown [79-81]. Gentamicin and/or ceftazidime caused histological changes as well as significant decrease in the body weights and urine output along with increase in protein and blood urea, serum creatinine, ESR, renal tissue levels of MDA and kidney weights in comparison with control rats. These changes were prevented by saffron [81].

#### Hexachlorobutadiene

Hexachlorobutadiene, is a potent nephrotoxic in rodents, which can cause degeneration, necrosis and regeneration in renal tubular epithelial cells [82]. Boroushaki et al. (2007) revealed that safranal (0.25 and 0.5ml/kg), has a protective effect against nephrotoxicity induced by hexachlorobutadiene in rats [82].

#### Cadmium

Cadmium (Cd) is an extremely toxic heavy metal used in industry. It is known to cause serious environmental and health effects including damage to renal and testis [83]. Asadi et al. (2014) showed that cadmium reduced sperm count, motility and vitality in comparison to control group. Saffron improved sperm parameters [84].

### Protective effects of saffron against chemical-induced hematological toxicity

#### Diazinon (DZN)

Hariri et al. (2011) showed that vitamin E, safranal (0.025 and 0.05 ml/kg) and crocin (50 mg/kg) restored the reduction of red blood cells, hemoglobin and hematocrit induced by diazinon. These agents also prevented the reduction in platelets and the increase in reticulocytes. Vitamin E, crocin and safranal did not inhibit the effect of diazinon on RBC cholinesterase activity [85].

### Protective effects of saffron against chemical-induced embryo toxicity

It is indicated that saffron aqueous extract (200 mg/kg) treated animals revealed improvement in maternal weight gain, embryolethality and bone ossification impaired by administration of AlCL_3_ (200 mg/kg) during the embryogenesis (6th to 15th day of gestation) period [86]. Although saffron’s usage in pregnancy can have some complication on the embryo. A study showed that high concentrations of the aqueous extract of saffron (0.2%) can produce embryonic abnormalities [87]. Moreover Moallem et al. (2013) reported crocin or safranal can induce embryonic malformations when administered in pregnant mice as evidenced by decrease in length and weight of fetuses and induction of minor skeletal malformations, mandible and calvaria malformations, and growth retardation [68].

### Saffron as a protective agent against its constituent

It was also shown that saffron could act as an antidote against its toxic constituent [88]. Safranal, the main component of *C. sativus* essential oil, is thought to be responsible for the unique odor of saffron [5,6]. Safranal has been shown protective effects against some drug- and chemical- induced toxicity [73,79,82,85]. However, this constituent itself also exhibited side effects [89]. According to Iranian Traditional Medicine (ITM), the usage of whole plant may reduce some adverse effects induced by plant containing toxic ingredients [90]. A study by Ziaee et al (2014) showed that the aqueous extract of saffron stigma could reduce the toxicity of safranal [88]. It was found that the co-treatment of safranal and saffron significantly reduced the mortality rate induced by safranal and improved significantly all toxic effects of safranal on biochemical parameters in acute and subacute toxicities. Therefore, the consumption of saffron as a whole plant could be considered as a valuable method to reduce safranal toxicity [88].

## Conclusions

In this review article, the different in vitro and animal studies summarized in order to discover the efficacy of saffron and its active constituents in protection against toxicities induced by natural or chemical toxins in different tissues.

According to the results of several important investigations, saffron and its active components act as an antidote in different intoxications induced by natural toxins including snakebites, mycotoxins and endotoxins.

Furthermore it is established saffron could act as an antidote for its constituent, safranal, which has more toxicity than the other constituents presented in saffron. Metals (Al, Be and cadmium), pesticides (DZN and PQ), acrylamide, benzo[a]pyrene and CCL4 are some examples of environmental and/or industrial chemical toxins which saffron could protect different tissues such as brain, cardiovascular, lung, kidney and liver against their toxicities. It is also documented this plant with wonderful power of therapeutic effects, exhibits protective effects against some chemical drugs such as antitumors (cisplatin, doxurobicin, cyclophosphamide and mitomycin), antibiotics (gentamycin and ceftazidime), analgesics (acetaminophen) which have organ toxicities especially in overdose. Some mechanisms including antioxidant, the modulation of cardiac, renal and liver enzymes, improvement in antioxidant defense systems, and the inhibition of apoptosis are involved in saffron antidotal effects.

In conclusion, based on the current review, saffron has an extensive spectrum of protective properties against toxicities induced by either natural or chemical toxins. As these findings have not yet been verified by clinical trials on humans, to establish the antidotal effects of saffron in human intoxications, human trials should be carried out.

## Abbreviations

AST: Aspartate transaminase
ALT: Alanine transaminase
ALP: Alkaline phosphatase
γ-GGT: Gamma glutamyl transpeptidase
GST: Glutathione transpeptidase
GSH-Px: Glutathione peroxidase
LDH: Lactate dehydrogenase
CK-MB: Creatine kinase MB
ROS: Reactive oxygen species
SOD: Superoxide dismutase
CAT: Catalase
AChE: Acetylcholinesterase
BuChE: Butyrylcholinesterase
MAO: Monoamine oxidase
DIC: Disseminated intravascular coagulation
MPO: Myeloperoxidase
3NPA: 3-Nitropropionic acid
IL-6: Interleukin-6
MCP-1: Macrophage chemo attractant protein-1
TNF-α: Tumor necrosis factor-α
iNOS: Inducible nitric oxide synthase
HO-1: Heme oxigenase-1
